# Tumor Diagnosis and Treatment: Imaging Assessment

**DOI:** 10.3390/tomography8030118

**Published:** 2022-05-30

**Authors:** Filippo Crimì, Federica Vernuccio, Giulio Cabrelle, Chiara Zanon, Alessia Pepe, Emilio Quaia

**Affiliations:** Institute of Radiology, Department of Medicine-DIMED, University of Padova, 35128 Padova, Italy; federica.vernuccio@aopd.veneto.it (F.V.); giulio.cabrelle@gmail.com (G.C.); chiara.zanon.5@studenti.unipd.it (C.Z.); alessia.pepe@unipd.it (A.P.); emilio.quaia@unipd.it (E.Q.)

At present, oncologic imaging is crucial for clinical decision-making. Imaging is used for cancer screening, diagnosis, staging, restaging and monitoring recurrence [1].

This Special Issue includes full research articles, case reports and reviews focused on oncological imaging to detect tumors and assess response after chemo-, radio-, or immuno-therapy. 

Scientific societies are promoting standardized reporting systems for diagnosis and screening to reduce the variability in the reports and improve the detection of small tumors. The standardized reports aimed to collect information about present and past illness of the patient, symptoms and risk factors, previous diagnostic investigations, together with a classification of the pathology of interest and imaging data of the detected lesions. In this way, the reports not only increase physicians’ ability to reach an accurate and standardized diagnosis for appropriate clinical management, but have become a tool of utmost importance for collecting data to be used in statistical analysis for research purposes [2]. In recent years, different Reporting and Data Systems (RADSs) have been published to standardize the reports of different tumors (BI-RADS, LI-RADS, PI-RADS, My-RADS, etc.) [3,4,5,6]. These criteria are continuously evolving and improving, since new imaging techniques have been developed, and it is necessary to increase the accuracy of the reporting systems as much as possible.

Multiple criteria have been proposed to evaluate the tumor response and metastases to neoadjuvant or adjuvant therapies (chemo-, radio- or immune-therapies), starting from the well-known RECIST criteria [7] and ranging to volumetric analyses and the evaluation of the image texture. Conventionally, imaging is qualitatively assessed, leaving much information that remains unexploited. A particularly difficult parameter to qualitatively assess is the tumors’ intralesional heterogeneity, which has been demonstrated to correlate with a worse outcome for the patients and more aggressive tumors [8]. Using textural analysis (TA), it is possible to elaborate the raw data that are provided by any technique (ultra-sound (US), computed tomography (CT), magnetic resonance imaging (MRI) and positron emission tomography (PET)), to obtain quantitative parameters that are not normally discernible to radiologists by eye, but that can provide a detailed characterization of any lesion [9]. TA can assess tumor heterogeneity by analyzing the relationship and distribution of pixel or voxel gray levels in the lesion area [10]. The TA is a non-invasive imaging tool with great potential to predict pathologic features, response to therapy and prognosis for many tumors [11]. In specific settings, the automatic or semiautomatic extraction of the textural features has shown similar or even stronger accuracy when detecting cancer compared to the qualitative evaluation of expert radiologists [12,13]. This technique provides new diagnostics pathways and, by means of integration with artificial intelligence algorithms in the elaboration of such large amount of data, could become a routinary application in the near feature. The correlation between radiomics data and clinical reports, pathology, histology and genetic information could provide a global view of the tumor biology [14].

Another field of research that has shown impressive results in oncological diagnostics is hybrid imaging, which is mainly driven by the recent introduction of the PET/MRI scanners. These imaging modalities have three main advantages compared to PET/CT [15]. First of all, compared to CT, MRI shows a higher contrast resolution for tissue characterization, which is particularly valuable for soft tissue, using specific multiparametric information techniques (e.g., mapping, diffusion-weighted imaging (DWI), dynamic contrast-enhanced (DCE)). Secondly, it is possible to obtain a better anatomical localization of the radio-tracers activity inside the solid organs in the fused PET/MR images, and profit by the tissue characterization. Finally, there is a significant reduction in exposure to ionizing radiation in PET/MRI compared to PET/CT, making PET/MRI more suitable for pediatric patients and young women. The combination of morphological, functional and metabolic information in the same test has raised this new technique as a possible “one-stop-shop” tool in oncological imaging that can aid in the diagnosis and clinical management of oncologic patients with a lower biological cost. In the clinical setting, PET/MRI was primarily used for prostate cancer, neuro-oncological diseases, soft-tissue sarcomas, colorectal cancer, pediatric malignancies and in cases of persistent clinical suspicion of abdominal malignancy with negative imaging findings [15,16].

Thus, standardized reports, TA and PET/MRI represent three of the main research fields in which oncological imaging has been developing in recent years. Still, the continuous improvements in radiological techniques are broadening the horizon for the applications of medical imaging in clinical routine.

This Special Issue collects papers that show how imaging can improve the early detection of tumors to hasten patients’ access to treatments and better evaluate their response. In the continuous improvement in chemotherapy and organ-saving approaches, it is of paramount importance to accurately assess the size of the residual tumor to determine the most appropriate treatment.
